# A blueprint for vitamin D fortification in sugar: stability and health impact

**DOI:** 10.3389/fnut.2025.1671785

**Published:** 2025-09-05

**Authors:** Hardinsyah Hardinsyah, Rahmawati Rahmawati, Ahmad Sulaeman, Fahrul Nurkolis

**Affiliations:** ^1^Department of Community Nutrition, Faculty of Human Ecology, IPB University, Bogor, Indonesia; ^2^Food Technology Program, Sahid University, Jakarta Selatan, Indonesia; ^3^State Islamic University of Sunan Kalijaga (UIN Sunan Kalijaga), Yogyakarta, Indonesia; ^4^Master of Basic Medical Science, Faculty of Medicine, Universitas Airlangga, Surabaya, Indonesia; ^5^Medical Research Center of Indonesia, Surabaya, Indonesia

**Keywords:** vitamin D, granulated sugar, food fortification, stability, health, 25-hydroxyvitamin D

## Abstract

Vitamin D deficiency is a global health concern, and fortifying widely consumed staples offers a scalable solution. Granulated sugar, accessible across socio-economic groups, shows promise as a novel vehicle for cholecalciferol delivery. Evidence indicates that vitamin D3 stability can be maintained through encapsulation and protective packaging, with over 90% potency retained under proper storage. Fortified sugar demonstrates good bioavailability, achieving absorption comparable to dairy products, and could increase serum 25(OH)D by 10–20 nmol/L at the population level. While offering low-cost impact, fortification requires careful dosing, quality control, and public education to mitigate risks of hypervitaminosis and prevent misperceptions that sugar itself is a health food.

## 1 Introduction

Food fortification is a widely recognized public health strategy aimed at enriching staple foods with essential nutrients to address widespread nutrient deficiencies (1–3). It offers a cost-efficient solution to target populations with poor adherence to supplementation. Given its broad consumption across all age and income groups, granulated sugar has been proposed as a potential vehicle for vitamin D fortification (4–7). Vitamin D plays a vital role in maintaining bone integrity and supporting immune function, yet its deficiency remains prevalent in many populations worldwide (8, 9). Adequate vitamin D levels help suppress excessive inflammation, promote regulatory T-cell development, and reduce the risk of autoimmune diseases such as multiple sclerosis, type 1 diabetes, and rheumatoid arthritis (10, 11). Current recommendations suggest maintaining serum 25-hydroxyvitamin D levels of at least 30 ng/mL (75 nmol/L), ideally 40–60 ng/mL (100–150 nmol/L), through diet, supplementation, and sunlight exposure to optimize bone and immune health (8, 12). Although clinical studies have not yet provided direct evidence on the health effects of vitamin D-fortified sugar, findings from other fortified food products indicate that, if properly implemented, sugar fortification may offer similar benefits (13–15). Before such an intervention can be effectively implemented, two key factors must be carefully evaluated: (1) the chemical stability of vitamin D when incorporated into sugar during storage and food processing, and (2) the biological and public health implications of consuming vitamin D-fortified sugar, including the nutrient's bioavailability and any potential health benefits or risks. This report explores these critical considerations using current scientific data and evidence.

## 2 Vitamin D stability in sugar

From a chemical standpoint, vitamin D exhibits relative stability under controlled conditions; however, it is susceptible to degradation when exposed to certain environmental stressors. Among the various forms of vitamin D (cholecalciferol, calcifediol, and calcitriol), only cholecalciferol is recommended for use in fortification and supplementation due to its superior stability, potency, and resistance to degradation during cooking and baking compared to others (16). Granulated sugar, due to its chemically inert nature, does not directly interact with vitamin D. As a result, the stability of vitamin D in fortified sugar largely depends on how the fortification is carried out and the conditions under which the product is stored. Scientific studies have identified several environmental factors that can reduce the integrity of vitamin D, including ultraviolet (UV) light exposure, air oxidation, elevated temperatures, and high humidity levels (17). Moreover, acidic conditions may induce structural changes in the vitamin molecule, potentially affecting its efficacy. The following section outlines the primary factors influencing the stability of vitamin D in sugar and other dry food matrices.

### 2.1 Ultraviolet light (UV)

Ultraviolet rays can damage vitamin D through photodegradation (photo-oxidation) to suprasterol I, superasterol II, and 5,6-transvitamin D3 with a quantum yield of 0.42 ± 0.1 (18). When vitamin D-fortified sugar is stored in clear or translucent containers exposed to sunlight or intense artificial light, the vitamin content can deteriorate significantly (19). This vulnerability has been observed in studies on vitamin D-fortified dairy products, where exposure to light caused a notable reduction in vitamin D levels. As a preventive measure, the use of opaque or UV-blocking packaging is strongly recommended for fortified food products (20). The same principle applies to vitamin D-enriched sugar. To preserve the vitamin's stability, it should be stored in sealed and dark containers that minimize light exposure during transportation and storage.

### 2.2 Oxygen and air exposure

Exposure to oxygen accelerates oxidative degradation of vitamin D, which is especially vulnerable in dry sugar lacking a lipid matrix (21). Airtight packaging and antioxidant incorporation can help protect stability and extend shelf life during storage and distribution (22, 23).

### 2.3 High temperature

Vitamin D is relatively heat-stable up to ~200 °C, but prolonged or high-temperature exposure accelerates degradation through isomerization (19). In fortified bread, 170 °C baking retains ~85% of vitamin D, while 200 °C reduces retention to 40–65% (21). For sugar fortification, low-temperature mixing preserves stability, though cooking or caramelization can cause partial losses. In the context of sugar, fortification is usually done through mixing at low temperatures; however, if fortified sugar is used in cooking (e.g., for caramelization or baking), a portion of vitamin D may be lost due to high heat.

### 2.4 Moisture

Moisture strongly influences vitamin D stability in dry food systems. Granulated sugar's low water activity helps limit oxygen exposure and preserve vitamin D, but high humidity causes clumping and accelerates degradation. Data from fortified wheat flour show that vitamin D_3_'s half-life is about 173 days under dry conditions (a_p_ 0.33, 25 °C), falling to 116 days in high humidity (a_p_ 0.93) and just 63 days at 45 °C (21). These findings underscore the importance of maintaining low humidity and cool storage to prolong vitamin D stability in fortified sugar (21, 24).

### 2.5 Acidic conditions

Although sugar is chemically neutral (pH ~7), vitamin D is sensitive to acidic environments, where it can undergo acid-catalyzed isomerization into biologically inactive forms. This effect is illustrated in vitamin D_2_-fortified rye bread, which, due to its lower pH, retained only ~73% of its vitamin D after baking compared to ~85% in less acidic wheat bread. The loss is attributed to the conversion of vitamin D_2_ into isomers like isotachysterol (21). While pure sugar does not pose an acid risk, interactions with acidic components such as fruit juices or sour food matrices could subtly reduce vitamin D's stability during use.

The mechanism of Vitamin D degradation have been identified in some pathways, which are photo-oxidation, free radical oxidation, and acid-induced isomerization (21, 25). Photo-oxidation is triggered when vitamin D absorbs light energy, leading to the formation of oxidized products. Free radical oxidation typically involves oxygen and trace metals, which break down the vitamin's side chain (21, 26). Acid-catalyzed isomerization alters the structure of the vitamin's double bonds, producing inactive forms like lumisterol or tachysterol. While these degradation products are non-toxic, they no longer retain biological activity. There is no report about sugar itself chemically react with vitamin D or accelerate its breakdown. Instead, environmental factors (light, oxygen, heat, and acidity) are the main contributors to instability. In this context, sugar serves as a relatively safe carrier, provided that storage conditions are well controlled.

The Strategies to Enhance Stability of vitamin D in fortified sugar, careful attention must be paid to processing methods and packaging by the manufacturer. Common fortification techniques involve incorporating vitamin D into a premix, which is then coated onto sugar crystals using surface coating or encapsulation technologies. Encapsulation is embedding vitamin D in microcapsules or nanoparticles offers added protection against oxygen and light exposure. Reviews suggest that encapsulation methods, along with using vitamin D_3_ (which is more stable than D_2_), improve retention in food matrices (27). Mixed micelles and nanoemulsions, especially those stabilized by surfactants such as Tween 80 and lecithin, help preserve vitamin D against degradation caused by temperature changes, light, and oxidation, maintaining over 70% retention after 1 month of storage at 4 °C (28–30). Water-dispersible formulations of vitamin D have also been developed to enhance uniformity and stability during mixing.

Packaging plays a critical role. Fortified sugar should be stored in airtight, opaque containers and kept in cool, dry environments (23). Studies have shown that with proper formulation and packaging, vitamin D3 can retain over 90% of its potency during storage in fortified products such as UHT milk, yogurt, and packaged juice over several weeks (19). In summary, vitamin D remains stable in fortified sugar when environmental stressors are controlled. No scientific evidence indicates that sugar alone degrades vitamin D; instability arises primarily from exposure to light, oxygen, heat, or acidic conditions. With optimized processing and storage, vitamin D-fortified sugar can maintain its nutritional value throughout distribution and shelf life.

## 3 The effect of fortifying sugar with vitamin D on health

### 3.1 Bioavailability of vitamin D in sugar

A key question is whether vitamin D added to sugar remains bioavailable upon consumption. In general, vitamin D fortification in foods has demonstrated good bioavailability, meaning the vitamin can be effectively released from the food matrix and absorbed in the small intestine (21). Studies have consistently shown that vitamin D3 from fortified products like milk (31), cheese (32–36), yogurt (37), bread (38) and orange juice (39) has comparable bioavailability to that from supplements. There is no evidence suggesting that sugar interferes with or deactivates vitamin D. When fortified sugar is consumed (e.g., dissolved in beverages or incorporated into recipes), the vitamin dissolves alongside the food or drink and is absorbed, particularly when dietary fats are present.

Since vitamin D is fat-soluble, optimal absorption occurs when consumed with fat. Pure granulated sugar contains no fat; thus, in extreme cases such as ingesting fortified sugar alone absorption may be suboptimal (40, 41). However, in real-world scenarios, sugar is typically consumed with other ingredients (e.g., milk in tea or desserts), which often provide enough fat to aid vitamin D absorption (20, 41). Even small amounts of fat can facilitate uptake in the gut. Research supports that lipid-based food matrices, such as dairy products, enhance both the stability and absorption of vitamin D. Bread fortified with vitamin D has shown potential to elevate serum 25(OH)D levels and reduce parathyroid hormone concentrations; however, additional research is required to validate its efficacy in the prevention or treatment of vitamin D deficiency (38). Therefore, vitamin D in fortified sugar remains bioavailable, especially when part of a balanced diet containing fats. Once absorbed, its metabolic fate is identical to other sources converted to 25(OH)D in the liver and utilized by the body as normal (13, 41).

### 3.2 Potential health benefits

Enriching sugar with vitamin D holds considerable potential for enhancing public health outcomes, especially in regions where vitamin D deficiency is widespread. Several key benefits include:

#### 3.2.1 Enhancing population-level vitamin D intake and status

Daily integration of vitamin D into sugar could markedly improve intake and reduce deficiency at the population level. Evidence from milk and margarine fortification shows significant rises in serum 25(OH)D—up to 17 nmol/L—and reductions in deficiency prevalence from over 90% to below 2% (42). Properly fortified sugar could achieve similar benefits, lowering risks of rickets, osteomalacia, and supporting overall bone and immune health.

#### 3.2.2 Reaching underserved and at-risk groups

Unlike individual supplementation, which depends on personal awareness and access, food fortification is universal by nature. Sugar is used across all socio-economic strata, meaning this method could reach individuals who may lack the resources or knowledge to obtain vitamin D supplements (43, 44). This is similar to the successful public health strategies that introduced iodine in salt and vitamin A in sugar (45). For instance, in Iran, daily consumption of a traditional drink (doogh) fortified with vitamin D not only raised serum 25(OH)D levels among participants but also improved glycemic control in individuals with type 2 diabetes (21). Such outcomes demonstrate how widely consumed fortified foods or beverages can serve as practical, large-scale health interventions.

#### 3.2.3 Long-term benefits for bone and immune health

Improved vitamin D levels across a population are associated with reduced prevalence of health problems related to its deficiency. Since vitamin D is crucial for calcium absorption, maintaining adequate levels supports bone mineralization, helps prevent osteoporosis, and lowers the risk of fractures in older adults (46–48). Beyond skeletal benefits, vitamin D is recognized for its broader impact on health, including modulation of immune function, reduction of inflammation, and potential protection against chronic diseases like cardiovascular disease, diabetes, autoimmune disorders, and certain cancers (8, 46, 49–51). While sugar fortification does not directly deliver these benefits, the resulting improvement in vitamin D status can indirectly lead to long-term positive health outcomes, such as decreased incidence of rickets, enhanced bone strength, and possibly stronger immune responses. Meta-analyses have indicated that national vitamin D fortification policies are linked with measurable declines in deficiency-related conditions and improvements in health indicators (27).

It is important to emphasize that the success of such an initiative depends on implementing proper fortification levels aligned with daily recommended intakes without surpassing upper safety limits and should be supported by nutrition education campaigns. Fortifying sugar with vitamin D does not make it a therapeutic product, but rather a functional food component aiding in daily micronutrient adequacy (13, 43, 44).

### 3.3 Potential risks and health perspectives

While fortifying sugar with vitamin D offers potential public health benefits, it also presents several challenges and risks that must be carefully considered:

#### 3.3.1 Risk of vitamin D toxicity (hypervitaminosis D)

Excessive intake of vitamin D can lead to hypercalcemia (elevated calcium levels in the blood) which may cause symptoms like nausea, fatigue, and even kidney damage (52). Although vitamin D toxicity is rare from regular food consumption, errors in the fortification process can be dangerous. A case reported in The Lancet described vitamin D poisoning due to table sugar that had been adulterated with doses thousands of times higher than recommended. Similarly, there was an outbreak of vitamin D toxicity when milk was mistakenly fortified with over 200,000 IU per liter, leading to poisoning in several individuals. These cases highlight the critical importance of quality control in the fortification process. To minimize risk, manufacturers must ensure that added vitamin D levels align with regulatory standards typically a few hundred IU per serving and that the distribution is homogeneous. When properly formulated and controlled, the risk of toxicity is extremely low. For reference, the tolerable upper intake level for vitamin D in adults is about 4,000 IU per day, and fortification levels are generally far below this threshold (53). However, without rigorous quality assurance, mass overdosing remains a serious concern, making strict regulatory oversight essential.

#### 3.3.2 Behavioral side effects (increased sugar consumption)

Fortifying sugar with vitamin D risks misleading consumers into viewing it as a “healthy” product, potentially driving higher intake despite sugar's link to obesity and diabetes. Market trends show micronutrient-enriched sugary foods can encourage excess calories, so any fortification program must be paired with clear public education that the goal is nutrient delivery, not promoting more sugar consumption (17, 54, 55).

#### 3.3.3 Unequal distribution of vitamin D intake

Sugar fortification relies on assumed uniform consumption, but intake varies widely. People with low sugar intake—such as those with diabetes or restrictive diets—may not receive enough vitamin D, while heavy consumers could get disproportionately more. Although overdose risk is low with proper dosing, this uneven distribution highlights the need to pair sugar fortification with complementary strategies to ensure adequate coverage for all groups (43, 56).

#### 3.3.4 Potential interactions with other nutrients

In some cases, fortifying sugar with multiple micronutrients can lead to interactions between vitamins. For example, in certain Latin American countries, sugar is also fortified with vitamin A. If both vitamins are added, their stability in the mixture must be verified. Some studies show that certain vitamins can affect each other's stability when combined. However, reports of significant interactions between vitamin D and other fortifying agents are rare. Vitamin D is generally stable and non-reactive when mixed with other vitamins and minerals. The key is to ensure that the vitamin D premix does not contain compounds that may accelerate degradation (13).

## 4 Integrative interpretation

**Figure 1:**
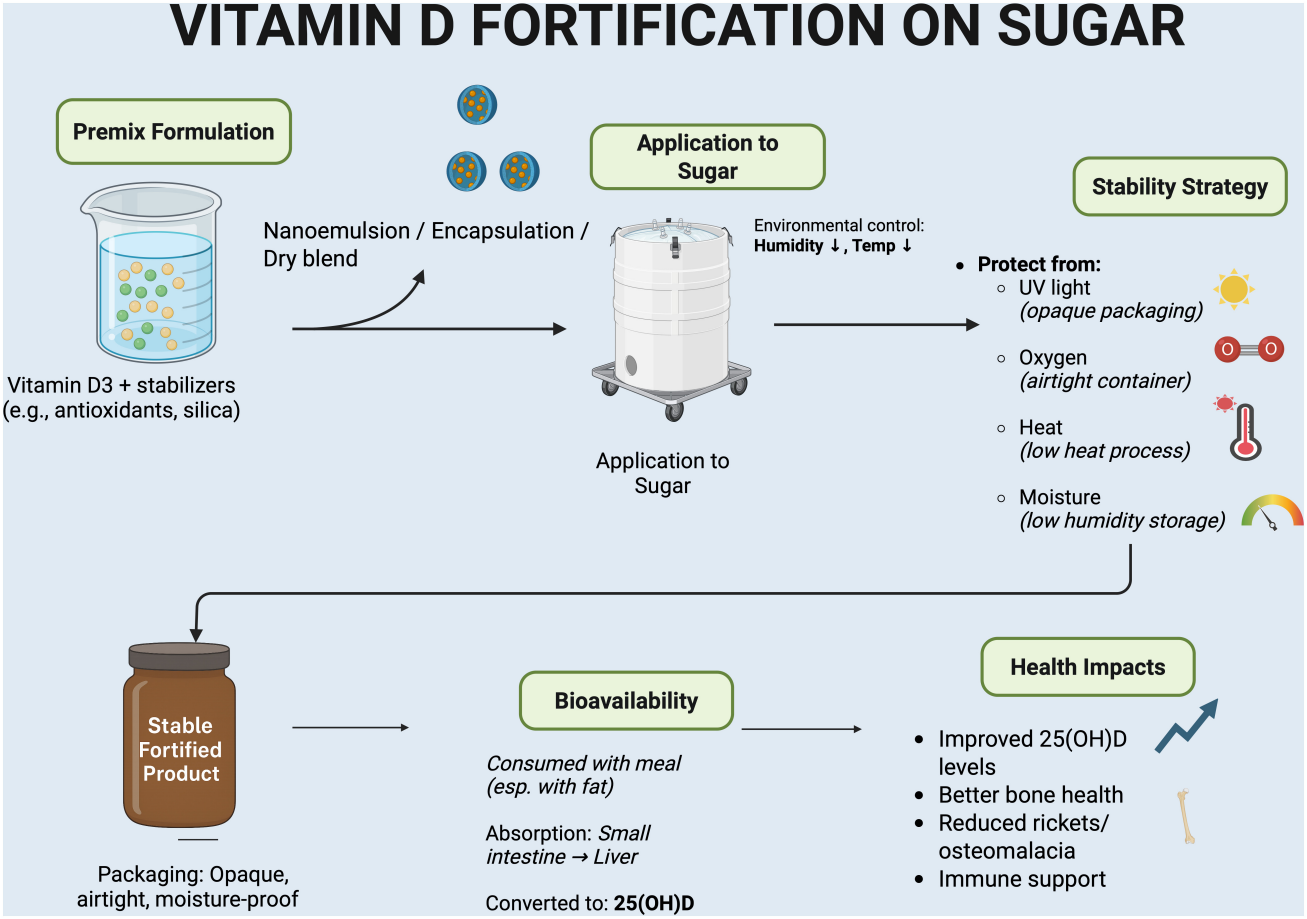
Schematic diagram of the vitamin D fortification process in granulated sugar.

Taken together, enriching sugar with vitamin D presents a promising nutritional intervention, provided it is executed with careful planning and rigorous oversight (Figure 1). The stability of vitamin D within sugar can be well maintained during storage, especially when destabilizing factors such as ultraviolet light, air exposure, heat, and humidity are effectively controlled (Figure 1). Sugar itself does not directly degrade vitamin D; in fact, under optimal storage conditions, over 90% of the vitamin can remain intact throughout the product's shelf life. Moreover, vitamin D delivered through fortified sugar remains bioavailable and has been shown to effectively elevate the body's vitamin D status, as evidenced by successful food fortification programs that have significantly increased population serum 25(OH)D levels (13, 18, 20).

On the other hand, safety considerations and public education must not be overlooked. The amount of vitamin D added should be carefully calibrated to ensure it is safe for daily intake across all age groups, with levels set well below toxic thresholds. A monitoring system for periodic testing of vitamin D content in fortified sugar should be established to prevent accidental overdosing. Additionally, it is crucial to communicate to the public that vitamin D-fortified sugar is not a license for excessive sugar consumption; sugar remains a food component that must be consumed in moderation for metabolic health. The primary goal of fortification is to enhance the nutritional value of sugar, not to encourage its increased intake (43, 57, 58).

## 5 Future directions, safety risks, and economic impact

Vitamin D deficiency remains highly prevalent in Indonesia, and fortification offers strong health and economic returns—up to US$27 gained for every US$1 invested, with European programs showing substantial cost savings. Cholecalciferol (D_3_) is more effective than ergocalciferol (D_2_), making it the preferred form for large-scale use. Future research should optimize sugar-based matrices for stability in tropical climates, and conduct long-term trials to evaluate impacts on serum 25(OH)D, fracture risk, and metabolic outcomes, alongside cost-effectiveness analyses across diverse regions. Safety remains critical: intakes above the tolerable upper level (4,000 IU/day) can cause adverse effects, requiring monitoring of serum calcium and vitamin D, particularly in vulnerable groups. Vehicle choice also matters, with sucrose favored over HFCS to avoid additional cardiometabolic risks. By leveraging Indonesia's existing sugar supply chains, fortification could reduce healthcare costs, improve productivity, and lessen the burden of metabolic and skeletal disease at scale.

## 6 Conclusion

Vitamin D–fortified sugar presents a practical alternative to milk, flour, or oils due to its universal consumption, stability, and ease of distribution. Its crystalline structure enables uniform dispersal of vitamin D, with fewer sensory or rancidity issues compared to oils. However, sugar's caloric load, link to caries and metabolic disease, and risk of vitamin D degradation or over-fortification demand cautious messaging and precise dosing. Future work should test bioavailability in human trials, assess consumer acceptance, evaluate storage stability, and model population-level health impacts. By weighing benefits and risks, this article highlights sugar's potential as a scalable fortification vehicle while guiding research and policy for safe implementation.
